# Miscellaneous

**Published:** 1892-07

**Authors:** 


### MISCELLANEOUS.

### WHAT SALT WILL DO.

**The common table salt is one of the most effective remedies for many ills.**

### FOR NASAL CATARRH.

**Make a weak brine of salt and tepid water and snuff up the nose, allowing it to run down the throat. If persistently used it will in a sufficient length of time cure the nasal catarrh.**

### CURE FOR A FELON.

**Common rock salt, dried in the oven and then pulverized, mixed with an equal part of spirits of turpentine. Keep a rag wet constantly with this preparation for twenty-four hours. It will surely cure a felon in that time.**

### RELIEF FOR NERVOUS HEADACHE.

**The simplest remedy known for this terrible trying affliction is a pinch of salt taken on the tongue and allowed to dissolve slowly, followed in about ten minutes with a drink of water. Salt, in its pure state, has virtues not to be scorned because it is an article within the humblest means.**

### THE FIRST UMBRELLA IN ENGLAND.

Jonas Hanway was the son of a storekeeper in the dockyard at Portsmouth, and on the death of his parents was bound apprentice to a merchant in Lisbon. When the term of his indentures had expired, he went to St. Petersburgh, where he became partner in a good house of business, and being desirous of opening up a trade with Persia, and also of penetrating into that land of mystery, journeyed thither, meeting with strange and wild adventures and enduring many hardships. But he picked up much information, which, on his return to England, he published; and he brought back with him rich experiences, a fair competence, and

—an umbrella ! Picture Jonas Hanway, with his plain honest face and his suit of broadcloth, walking through the streets of London, the first man who ever used an umbrella ! People stood and stared, boys jeered and hooted, and some thought him mad, while others only laughed at him for being eccentric. But Jonas had a purpose ; he found the umbrella useful in wet weather to shield him from the rain, and in summer to keep off the sun, and at other times to serve him as a stick, and wherever he went he persistently carried this curiosity, until people got accustomed to see it. After a time, on wet days, Jonas was not the only man to use it ; one after another took to the "ridiculous" umbrella until at last a new trade was originated, and to-day it is the source of a livelihood to thousands.

---

To Cure Warts.—A very simple remedy for the cure of warts is the following. Pass a clean, bright new pin through the wart, and hold it so you can apply one end of the pin to the flame of a lamp ; hold it there until the wart fries under the action of the heat. A wart so treated will take final leave. A wart with a slender root may be easily destroyed by fastening around it a silk thread or horsehair. After it drops off the roots should be touched with caustic to prevent it growing again. Hard warts should be cut smoothly off with a knife or sharp scissors, and then caustic applied to their roots to destroy them. Warts may also be cured by touching repeatedly with lunar caustic, blue vitriol or chloride of zinc.

Apparition at the Moment of Death.—A woman who lived with her husband and their little girl in a village some four miles distant from our house, came to assist in house cleaning. For convenience sake she slept in the house. Late one evening she went to fetch water from a well about fifty yards from the house. To the astonishment of the servants she presently rushed back, pale and trembling, to say that her little girl had appeared to her in her nightdress, holding out her arms to her. She felt sure something had happened, for when she called to her child, and ran to meet her, the figure vanished. She insisted on going home at once, and the servants vainly tried to persuade her to remain till morning, and that she had only imagined the appearance. Nothing could induce her to delay her return, and on being informed of the circumstances, we desired a groom to drive her home in a dog-cart. At a short distance from the village they met the woman's husband on his way to tell her that their little girl was dead. She had fallen from a window, and had died at the time her mother had seen the apparition.—*W., in Light.*

Minute Wonders of Nature.—Human hair varies in thickness from the 250th to the 600th part of an inch The fibre of the very coarsest wool is only the 500th part of an inch in diameter while in some species of the sheep it takes 1500 of their hairs laid side by side to cover an inch on the rule. The silk worm's web is only the 5300th part of an inch in thickness, and some of the spiders spin a web so minute that it would take 60,000 of them to form a rope an inch in diameter ! A pound's weight of spider's web of this size would reach around the world and then leave enough to reach from New York to San Francisco. A single grain of musk has been known to perfume a room for twenty years. At the lowest computation that grain of musk must have been divided into 320,000,000,000,000 particles, each of them capable of effecting the olfactory organs. The human skin is perforated by at least 1000 holes in the space of each square inch. For the sake of argument, say there is exactly 1000 of these little drain ditches to each square inch of skin surface. Now estimate the skin surface of the average sized man at sixteen square feet and we find that he has 2,304,000 pores.

ORIGIN OF OX-TAIL SOUP.—The exiles who took refuge in London at the time of the French Revolution met the poverty and hardships of their lot with much courage. They never begged, and it was often difficult to induce them to accept the funds subscribed for their assistance.

The women did not accept the partially worn and soiled clothing of wealthy and charitably inclined ladies, as most women in their condition would have been glad to do, but managed with the cheapest materials to dress neatly and tastefully.

Their necessities developed an inventive spirit. The records of the London Patent Office at the beginning of the eighteenth century have on every page such names as Blondeau, Dupin, Cardonel, Gastineau, Leblond and Courant. How ingenious they were in utilizing the most unpromising of materials is shown by their invention of a now famous dish.

When the London butchers slaughtered their beef they were accustomed to throw away the tails with the refuse. The French women had the bright idea of buying them, since they could get them for next to nothing, and making soup of them. And thus they gave to England the popular ox- tail soup, which loyal Englishmen now consider an essentially national dish.

OLD JOKES.—Is there really any new story under the sun ? Shortly after the death of Spurgeon an anecdote was current relating that once, when the great popular divine was preaching from the text, "I can do all things," he thumped his pulpit and said, " Aye, can ye, Paul ? I'll bet ye a half crown o' that ! But, stop ! let's see what else Paul says : 'I can do all things through Christ which strengtheneth me ;' aye, so can I, Paul ; I draw my bet." This identical anecdote is told in Parton's "Life of Horace Greeley," of a minister in the New England community during Queen Anne's reign, except that the word "dollar" is substituted for "half crown."

HEAT WITHOUT FIRE.—French chemists, says the *Aluminum Age*, have demonstrated that it is possible to produce heat without fire, and the discovery is to be utilized on the railways and street cars of the country. The device consists simply of a block of acetate of soda, which is plunged into hot water. As it solidifies after the immersion it gives forth as much heat as a coal fire for the space of five or six hours. There is no danger of fire from the use of the substance, and as the same fuel can be used a score of times, its cheapness will be a great recommendation with many managers of corporations.

GRUEL FOR THE VERY DELICATE.—A heaping tablespoonful of pearled tapioca to one quart of water. Wash the tapioca and put it into the water cold and let it come to the boiling point slowly. Let it boil gently until the tapioca is very soft. Strain it and add a little salt and cream if the patient can take it. This gruel is relished and has proved beneficial where all other nourishment has been rejected. In case of bowel troubles the addition of raisins, proportion of a tablespoonful to a cup of milk, cooked in the milk is of value.

SERVANT GIRL PROBLEM.—The servant girl problem has been solved on the Pacific coast where 15,000 Japanese are employed in that capacity. As yet the opposition to them is quite faint. The Japanese dress like Americans, drink like Americans, and are willing to vote early and often, if given half a chance.

REV. PLINK PLUNK ON FORTUNES.—Ebery man am de architect ob his own fortune, deah breddern, but the Vanderbilts an' Goulds doan need to fear any competition from de man dat hez been pervided by Fate with nuttin' but a pair o' hands an' a shovel or a hod to draw his plans wid.

